# Using alternatives to the car and risk of all-cause, cardiovascular and cancer mortality

**DOI:** 10.1136/heartjnl-2017-312699

**Published:** 2018-05-21

**Authors:** Jenna Panter, Oliver Mytton, Stephen Sharp, Søren Brage, Steven Cummins, Anthony A Laverty, Katrien Wijndaele, David Ogilvie

**Affiliations:** 1 MRC Epidemiology Unit, University of Cambridge, Cambridge, UK; 2 UKCRC Centre for Diet and Activity Research (CEDAR), University of Cambridge, Cambridge, UK; 3 Department of Social and Environmental Health Research, London School of Hygiene and Tropical Medicine, London, UK; 4 Public Health Policy Evaluation Unit, School of Public Health, Imperial College London, London, UK

**Keywords:** cardiac risk factors and prevention, hypertension, coronary artery disease, epidemiology, stroke

## Abstract

**Objective:**

To investigate the associations between using alternatives to the car which are more active for commuting and non-commuting purposes, and morbidity and mortality.

**Methods:**

We conducted a prospective study using data from 3 58 799 participants, aged 37–73 years, from UK Biobank. Commute and non-commute travel were assessed at baseline in 2006–2010. We classified participants according to whether they relied exclusively on the car or used alternative modes of transport that were more active at least some of the time. The main outcome measures were incident cardiovascular disease (CVD) and cancer, and CVD, cancer and all-cause mortality. We excluded events in the first 2 years and conducted analyses separately for those who regularly commuted and those who did not.

**Results:**

In maximally adjusted models, regular commuters with more active patterns of travel on the commute had a lower risk of incident (HR 0.89, 95% CI 0.79 to 1.00) and fatal (HR 0.70, 95% CI 0.51 to 0.95) CVD. Those regular commuters who also had more active patterns of non-commute travel had an even lower risk of fatal CVD (HR 0.57, 95% CI 0.39 to 0.85). Among those who were not regular commuters, more active patterns of travel were associated with a lower risk of all-cause mortality (HR 0.92, 95% CI 0.86 to 0.99).

**Conclusions:**

More active patterns of travel were associated with a reduced risk of incident and fatal CVD and all-cause mortality in adults. This is an important message for clinicians advising people about how to be physically active and reduce their risk of disease.

## Introduction

Physical activity, including less vigorous forms of physical activity such as walking and cycling, reduce the risk of cardiovascular disease (CVD).^1^ Despite the knowledge of its benefits, levels of activity are still low in many countries.^2^ With increasingly sedentary occupations and busy lives, many people have little time for leisure time physical activity. Activity as part of a journey, such as the commute or for transport in general, offers a comparatively easy way to integrate exercise into daily life.^3^

Prospective observational studies have shown associations between walking or cycling to work and health, principally through a reduced risk of cardiometabolic disease.^4–6^ While there is a good scientific rationale for focusing on walking or cycling to work due to its regular nature, for many comparatively car dependent populations, walking or cycling the entire journey is impractical because of the distances involved. For example, in the UK, only 17% of adults live within easy walking distance (2 km) of work and only 35% live within easy cycling distance (5 km).^7^ However, it is possible to incorporate more physical activity into journeys without completely replacing motor vehicle use—for example, by using public transport, or walking or cycling parts of longer journeys made by car. These travel patterns involve more physical activity than exclusive car use and can add up, over the course of a typical working week, to a substantial amount of activity.^8 9^ These travel patterns are prevalent in some, particularly urban and peri-urban, populations^10^ and are likely to be more achievable for many people, but have been rarely studied.^9^ In addition, with increases in home and remote working combined with an ageing population,^11^ an increasing proportion of adults are less likely to make regular commutes. Much research is focused on the benefits of active commuting but the potential health gains associated with non-commuting travel are less well known.

We aimed to extend previous research by using data from a large epidemiological cohort to investigate prospective associations between more active patterns of travel relative to exclusive car use and cardiovascular disease (CVD), cancer and all-cause mortality in the general adult population.

## Methods

### Study population and sample

We used data from UK Biobank, a national population based study of 5 02 639 men and women, aged 37 to 73 years.^12 13^ Potential participants were selected through population based registers of patients registered with the National Health Service (NHS) from across England, Scotland and Wales. Those living within 35 km of any of 22 assessment centres were invited. At baseline (March 2006–July 2010), participants reported information on sociodemographic characteristics, physical activity and health conditions. All participants provided informed consent.

**Figure 1:**
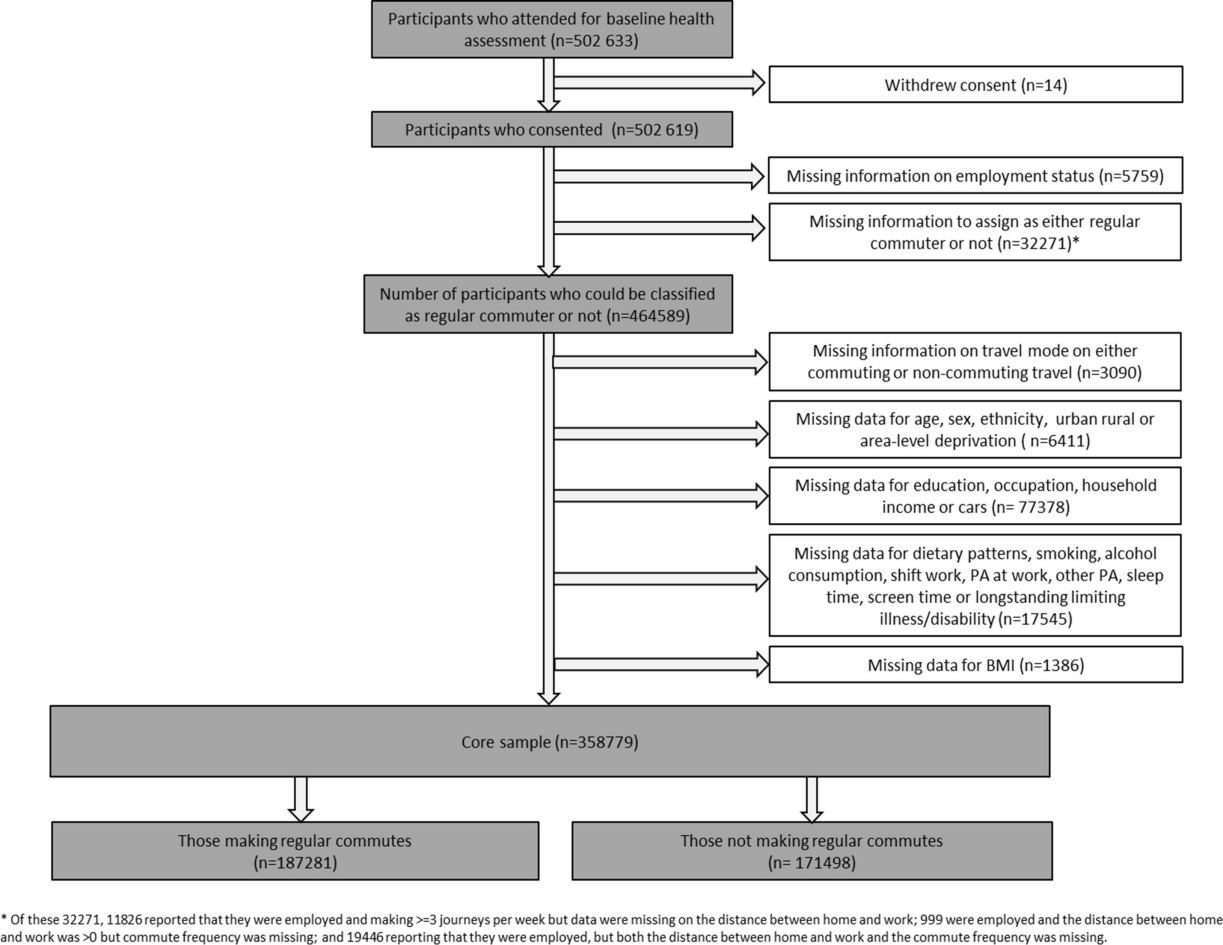
Sample of UK Biobank participants for analysis.

As there were some differences in demographic and health characteristics between those who commuted regularly and those who did not, we stratified our sample. We defined regular commuters as those participants who reported being employed, travelled to work at least 3 times/week and reported a home to work distance of greater than zero. Those not regularly commuting therefore comprised those who were not employed (eg, retired or unemployed), along with those who were employed but reported either travelling to work <3 times/week or a home to work distance of zero miles. We chose this definition as those working part time or commuting only part of the week constituted a small proportion of the total and were more similar to those who were not regularly commuting. Participants with missing information on employment status, commute frequency or distance were excluded (figure 1).

### Exposures

#### Commute travel

Participants in employment were asked "What types of transport do you use to get to and from work?" Six response options were given: car/motor vehicle, public transport, cycle, walk, ‘none of the above’ and ‘prefer not to answer’. Participants could select more than one response.

Using these responses, we divided participants into two behavioural patterns or ‘phenotypes’: (a) those who reported exclusive use of the car and (b) those who reported any other travel pattern (‘more active patterns of travel’)—that is, including some walking, cycling or public transport, either alone or in combination with the car. Participants who reported ‘none of the above’ or ‘prefer not to answer’ were excluded.

#### Non-commute travel

All participants were asked "In the last 4 weeks, which forms of transport have you used most often to get about?", with occupational travel specifically excluded. The same response options were provided as for the question on commuting. We classified these responses in the same way.

#### Commute and non-commute travel

In addition, we classified regular commuters into one of four categories according to whether they reported exclusive car use for commuting, non-commuting travel, both or neither.

### Outcomes

We studied five main outcomes: incident and fatal CVD (International Classification of Disease 10th revision, codes I20–25 for ischaemic heart disease and I60–69 for cerebrovascular disease), incident and fatal cancer (excluding all skin cancer (melanoma and other malignant neoplasms) C43–44) and all-cause mortality. In addition, we studied four other outcomes: incident and fatal colon cancer (C18) and incident and fatal breast cancer (C19), with which a lack of physical activity has been shown to be specifically associated.^14 15^ To minimise the potential effects of reverse causation, we excluded all participants with new events in the 2 year period after baseline assessment. Outcomes were identified by linkage to hospital records, the national cancer registry and death certificates. Censoring dates for these datasets differed and differed in different regions but all were complete up to 3 November 2015. For example, hospital admission data for England were available up to 31 March 2015 but for Wales it was later (29 February 2016).

### Covariates

Data from the baseline questionnaire were used to assess age, sex, ethnicity, highest educational qualification, occupation, household income, access to a car, dietary intake (through measures of consumption of fruit and vegetables), alcohol consumption, smoking status, occupational and recreational physical activity, shift working, sleep and screen time, longstanding illness/disability, medical conditions (high blood pressure, diabetes) and medication usage. Height and weight were measured at the assessment centre and used to compute body mass index. Area level indices (Townsend score of deprivation and urban/rural status) were derived from home postcodes.

### Statistical analyses

We used Cox regression to estimate the associations between more active patterns of travel and the hazard of each outcome. We made progressive adjustments to account for potential confounders (model 1: demographic and geographical characteristics; model 2: individual socioeconomic characteristics; model 3: other behaviours; model 4: other health conditions) restricting all models for a given outcome to participants with complete data for all covariates in model 4. Full details are provided in online supplementary table A1. For all outcomes, individuals with prevalent conditions were excluded (eg, for CVD mortality, those with prevalent CVD were excluded). The proportional hazards assumption was assessed using log–log survival plots, and graphical checks suggested the assumptions were reasonable.

10.1136/heartjnl-2017-312699.supp1 Supplementary file 1

For each of the main outcomes in regular commuters, we tested interactions between exposure and car access (none, 1, 2 or more) and home to work distance (<3 miles vs ≥3 miles). We chose these categories based on the prevalence of exclusive car use and distances reported in our sample. These characteristics may limit available travel options, be socially and spatially patterned, and thereby moderate the associations observed.

### Sensitivity analyses

Given the limited number of events observed, we undertook a sensitivity analysis excluding only participants with events in the first year (rather than 2 years).

## Results

### Sample characteristics and travel patterns

In total, data from 358 799 participants were included in the analysis. Those included were more likely to report at least degree level education, higher occupational status and higher household incomes, and to engage in higher levels of physical activity than those who were excluded (see online supplementary table A2). Of those included in the analysis, 187 281 were regular commuters (mean age at baseline 52.1±6.8 years) and 171 498 were not (mean age 60.7±6.9 years) (table 1). Regular commuters tended to be younger and healthier and to report a higher household income than those who did not regularly commute. Approximately two-thirds of commuters relied exclusively on the car to travel to work, with more active travel patterns being more frequently reported for non-commuting travel (table 2). While 81.7% of regular commuters and 77.3% of other participants reported using the car at least some of the time for non-commuting travel, 22.4% and 36.5%, respectively, reported some public transport use, and 44.9% and 52.5%, respectively, reported some walking. Cycling was less prevalent, being mentioned by 8.5% and 7.0% of regular commuters for commuting and non-commuting travel, respectively, and by 4.8% of other participants.

10.1136/heartjnl-2017-312699.supp2 Supplementary file 2

**Table 1:** Characteristics of the sample at baseline

	Regular commuters (n=1 87 281)		Not regularly commuting (n=1 71 498)	
	Mean	SD	Mean	SD
Follow-up time (years)	7.0	0.9	6.9	1.1
Age (years)	52.1	6.8	60.7	6.9
Body mass index (kg/m^2^)	27.2	4.7	27.5	4.8
Weekly time spent walking for leisure (min)	77.3	124.3	123.2	180.2
Weekly time spent in strenuous sports (min)	20.1	71.7	13.3	65.9
Weekly time spent in other exercises (min)	61.8	110.5	66.8	129.8
Weekly time spent in DIY activities (min)	92.1	209.4	134.4	276.6

	%	N	%	N
Sex				
Women	50.9	95 294	53.6	91 890
Men	49.1	91 987	46.4	79 608
Smoking status				
Never	58.0	1 08 580	50.9	87 357
Previous	31.4	58 726	39.3	67 448
Current	10.7	19 975	9.7	16 693
Ethnicity				
White	94.9	1 77 654	96.9	1 66 167
Non-white	5.1	9627	3.1	5331
Education				
University degree	39.2	73 396	30.9	52 967
A levels	35.4	66 322	31.2	53 518
GCSE or equivalent	16.7	31 310	16.6	28 453
None	8.7	16 253	21.3	36 560
Residential status				
Urban	86.9	1 62 711	84.1	1 44 158
Town and fringe	6.6	12 428	7.7	13 155
Rural	6.5	12 142	8.3	14 185
Occupation				
Managerial/professional	60.0	1 12 389	22.1	37 888
Administrative/skilled trades	22.5	42 227	7.3	12 495
Professional/customer services	8.6	16 175	3.1	5349
Operatives/labourers/other	8.8	16 490	2.6	4471
Not applicable (eg, retired)	0.0	0	64.9	1 11 295
Income				
<£31 000	30.2	56 640	65.2	1 11 739
£31000–<£52 000	32.3	60 578	19.9	34 169
≥£52 000	37.4	70 063	14.9	25 590
Number of cars owned				
0	5.8	10 769	9.7	16 639
1	37.0	69 216	47.0	80 591
2 or more	57.3	1 07 296	43.3	74 268
Shift work				
None	83.2	1 55 825	97.4	1 67 036
Day only	8.2	15 373	1.2	2101
Includes nights	8.6	16 083	1.4	2361
Physical activity at work				
Not applicable	0.0	0	80.7	1 38 378
Manual	12.4	23 282	1.8	3052
Standing/walking/some manual	30.1	56 311	5.5	9440
Light sedentary	22.9	42 819	4.1	7072
Sedentary	34.6	64 869	7.9	13 556
Longstanding limiting illness or disability				
No	75.6	1 41 669	60.9	1 04 436
Yes	24.4	45 612	39.1	67 062
Any medication used?				
No	82.2	1 53 968	63.5	1 08 827
Yes	17.8	33 313	36.5	62 671

**Table 2:** Exclusive use of the car in non-commuters and commuters

Travel patterns	Sample (% (N))	Mean (SD) follow-up time
**Regular commuters**		
Commuting		
Relying exclusively on the car	63.8 (119394)	7.0 (0.9)
More active patterns of travel	36.2 (67668)	7.0 (0.9)
Non-commuting		
Relying exclusively on the car	45.1 (84347)	7.0 (0.9)
More active patterns of travel	54.9 (102473)	7.0 (0.9)
Commuting and non-commuting travel		
Exclusive use of a car	37.5 (69824)	7.0 (0.9)
Exclusive use of a car for commuting, more active patterns of travel for non-commuting	26.3 (49236)	7.0 (0.9)
More active patterns of travel for commuting, exclusive use of a car for non-commuting	7.7 (14450)	7.0 (0.9)
More active patterns of travel for commuting and non-commuting	28.5 (53120)	7.0 (0.9)
**Not regular commuters**		
Non-commuting		
Relying exclusively on the car	34.5 (59143)	6.9 (1.1)
More active patterns of travel	65.5 (112073)	6.9 (1.0)

### Associations with main outcomes

**Figure 2:**
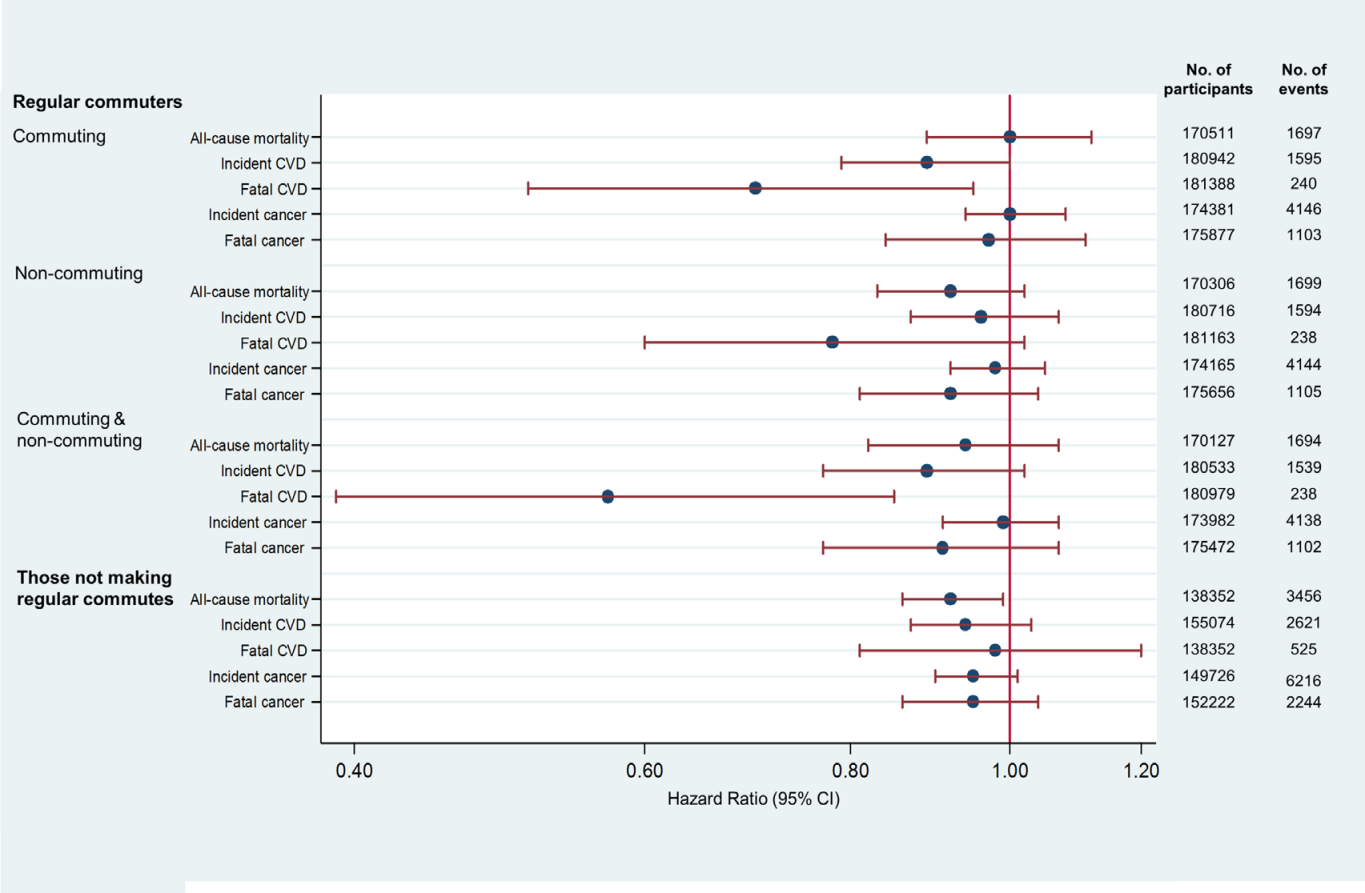
Maximally adjusted HRs for more active patterns of travel (compared with exclusive car use), and all-cause mortality, incident and fatal cardiovascular disease (CVD) and cancer for regular commuters and those not making regular commutes. The HR values for commuting and non-commuting travel are for commuters who use more active patterns of travel at least some of the time relative to commuters who rely exclusively on the car for both commuting and non-commuting travel.

Figure 2 shows the maximally adjusted associations (model 4) between more active patterns of travel and outcomes, and tables A3–A5 in the online supplementary material show the breakdown of progressive adjustment (models 1–4).

#### Regular commuters

Among regular commuters, more active patterns of travel for commuting were associated with estimated reductions of 11% in incident cases and 30% in fatal cases of CVD in models adjusted for demographic and socioeconomic characteristics, physical activity and dietary behaviours, and other health conditions (HR 0.89, 95% CI 0.79 to 1.00 and HR 0.70, 95% CI 0.51 to 0.95, respectively) (figure 2 and online supplementary table A3). More active patterns of travel for commuting were not significantly associated with incident or fatal cancer or all-cause mortality, and were not significantly associated with any of the outcomes for non-commuting travel (figure 2 and online supplementary table A3). However, dual exposure of more active patterns of commuting and non-commuting travel was associated with an estimated 43% reduction in fatal CVD events compared with exclusive car use for both types of travel in maximally adjusted models (HR 0.57, 95% CI 0.39 to 0.85) (figure 2 and online supplementary table A4). This dual exposure was also associated with a reduction in incident CVD in model 3, but the association was no longer significant in the maximally adjusted model which included other health conditions (model 4).

#### Those not making regular commutes

Among those not making regular commutes, more active patterns of travel were associated with an estimated 8% reduction in all-cause mortality in maximally adjusted models (HR 0.92; 95% CI 0.86 to 0.99) (figure 2 and online supplementary table A5). Associations for incident CVD and incident and fatal cancer were no longer significant in maximally adjusted models.

### Associations with other outcomes

There were no significant associations with breast or colon cancer incidence or mortality in any models (see online supplementary table A6).

### Sensitivity analyses and interactions

After relaxing the exclusion criteria such that only participants with events in the first year were excluded, the associations observed were of similar magnitude to those observed in the main analyses, with confidence intervals tending to be slightly narrower. Two associations became significant in regular commuters, for whom more active patterns of non-commuting travel were now associated with a lower risk of CVD and all-cause mortality (HR 0.76, 95% CI 0.59 to 0.98 and HR 0.91, 95% CI 0.83 to 1.00, respectively).

We found no evidence that any of the associations between more active patterns of travel and the five main outcomes were moderated by distance to work or car access (all P>0.01).

## Discussion

### Principal findings

Although not all associations were significant, the general pattern of our results indicates that, irrespective of other physical activity, more active patterns of travel, compared with exclusive car use, were associated with reductions in risk of incident and fatal CVD and all-cause mortality. Of note, in regular commuters, more active patterns of travel were associated with a reduced risk of both incident (11%) and fatal (30%) CVD; the reduction in CVD mortality was increased to 43% among those who used more active patterns for non-commuting travel. The latter exposure was also associated with a significant, albeit smaller (8%), reduction in all-cause mortality among those who were not regular commuters.

### Strengths and limitations

Strengths of this analysis include use of a very large multicentre general population dataset, a focus on feasible travel choices for commuting and non-commuting travel, and the linkage to objectively ascertained morbidity and mortality outcomes using national datasets. Our analysis extends previous research^6^ in important ways. These include more stringent exclusion of prevalent conditions, and incident cases occurring in the first 2 years; adjustment for a more comprehensive set of potential individual level confounders and other covariates, ranging from markers of socioeconomic position to behavioural characteristics (sleep, diet and other physical activity) and mostly self-reported health conditions; and consideration of non-commuting travel as an important exposure alongside the more frequently researched activity of commuting. In general, the progressive adjustment indicated that the magnitude of the associations were very similar (even if some results became non-significant). In combination, these approaches are likely to have reduced but not eliminated the risks of reverse causation, residual confounding and a healthy worker effect, any of which might lead to an overestimation of the true effects. UK Biobank participants are less ethnically diverse and healthier than the general UK population,^14^ and a substantial number gave insufficient information on key variables for them to be included in analysis. Participants who were excluded from the analysis also tended to report lower levels of physical activity, lower occupational classification and lower educational attainment than those who were included. While this admittedly limits the generalisability of some of our descriptive statistics to the national population, there is no particular reason to believe that our results are not generalisable in principle. Our analyses assume that travel patterns remain relatively stable over follow-up. We did not have information about changes in activity from the entire cohort, but repeated measures in less than 2% of our sample 4 years after baseline indicated that patterns of commuting remained very stable for the majority.^16^

### Comparison with other research

Our results are consistent with previous research suggesting that replacing exclusive car use with more active travel patterns may be beneficial for health.^17^ Of all the outcomes investigated, our results for incident and fatal CVD in regular commuters appear the strongest. The findings of a previous systematic review focused on active commuting and cardiovascular disease,^5^ as well as those of more recent studies, are somewhat inconsistent: some report positive (protective) associations for incident or fatal CVD,^5 18^ while others report null associations^19–21^ or mixed associations.^22 23^ However, given that our sample is substantially larger than that used in all but one of these previous studies,^5^ we suggest that our results shift the overall balance of evidence to a position that more clearly supports the potential contribution of active travel to the primary prevention of CVD in commuters. This is supported by other epidemiological evidence linking cardiovascular outcomes with physical activity in general,^24^ and linking active commuting and regular physical activity with plausible biological mechanisms, such as blood pressure reduction and anti-inflammatory effects.^6^

We also found that more active patterns of travel were associated with a reduced risk of all-cause mortality among those not regularly commuting. This result is in line with a meta-analysis^25^ which found that walking and cycling for either commuting or recreation were associated with reduced all-cause mortality. While the associations for more active travel with mortality have not previously been investigated in non-commuters, a systematic review examined the association between walking and cycling and mortality.^17^ In that review, of the five studies examining associations between active travel and all-cause mortality, only one found a significant association, which was observed for cycle commuting (walking on the commute was not examined in that study).

In terms of cancer outcomes, the associations we observed, although protective, were small and non-significant. Relatively few studies have described the associations between active travel and risk of incident or fatal cancers.^26^ Our non-significant findings may reflect the small numbers of cases of breast and colon cancer (the cancers with which physical activity in general appears to be most strongly associated) and the short follow-up period relative to the aetiological time period of cancer development.

### Implications for policy and practice

Taken together and in the light of existing evidence, our findings provide further support for a hypothesis that more active patterns of travel for both commuting and non-commuting purposes may be associated with significant reductions in CVD and all-cause mortality. This is an important message for clinicians advising people about how to be physically active and reduce their risk of disease. We also found no evidence that these associations were moderated by car access, which could be explained by the heterogenous nature of the group who did not rely on car use, but it may also suggest that the benefits are available to all, irrespective of car access or distance to work.

Demographic and technological trends in countries such as the UK are thought likely to result in a reduced requirement for commuting over time and a dispersal of older people towards more rural areas,^11^ both of which will increase the importance of non-commuting travel. Interventions that encourage people to reduce their car use in favour of making more use of public transport, walking, cycling, or combinations thereof, may be more widely applicable than efforts to promote walking or cycling in particular, especially among people whose circumstances preclude, for example, cycling all the way to work, or giving up the car completely in a rural area. Our own previous research has highlighted the potential health gains associated with integrating walking or cycling stages into longer journeys by car or public transport,^9^ a target for public health intervention also supported by recommendations from the National Institute for Health and Care Excellence,^27^ the UN and the WHO.^28^

### Implications for future research

Longer term or more rigorous longitudinal analysis could investigate in more detail the extent to which changes in travel behaviour result in individual health benefits. Cohorts such as UK Biobank provide the opportunity to follow-up large numbers of people at regular intervals (not just at baseline) over a longer period of time, and the accrual of more cases of disease over time will increase the power to detect associations that may not have become apparent to date. Collecting more detailed information about the frequency, duration and modal composition of trips, whether in this cohort or other future studies, would enable more definitive investigation of these associations and the extent to which they are modified by car access, distance or other factors.

**Key messages**

**What is already known on this subject?**

- Physical inactivity is an important risk factor for cardiovascular disease.
- Current clinical practice guidelines recommend physical activity, although the benefits of active travel on mortality and morbidity are still unclear.

**What this study adds?**

- We examined the association of active travel with mortality and morbidity in a cohort study.
- More active travel patterns were associated with significant reductions in cardiovascular disease (CVD).
- Those who used more active modes of travel for commuting and non-commuting purposes also had an even lower risk of fatal CVD.
- Among those who were not regular commuters, more active travel was associated with a lower risk of all-cause mortality.

**How might this impact on clinical practice?**

- This is important for clinicians advising people about how to be physically active and reduce their risk of disease.
