# Supplementary material for: Using alternatives to the car and risk of all-cause, cardiovascular and cancer mortality
Source: Heart. 2018 May 21;104(21):1749–55. doi: 10.1136/heartjnl-2017-312699 (PMC6241630; doi:10.1136/heartjnl-2017-312699)
Supplement: Supplementary file 1 [file heartjnl-2017-312699supp001.docx]

**Additional file 1: Additional methods**

**Table A1: Summary of co-variates adjusted for and exclusions applied in hazard models for five main outcomes**

|  | **All-cause mortality** | | **Incident CVD** | **CVD mortality** | **Incident cancer*** | **Cancer mortality*** |
| --- | --- | --- | --- | --- | --- | --- |
| **Exclusions** | | Deaths within first 2 years of follow-up are excluded.  Individuals with prevalent heart disease (including angina)/stroke (based on self-report or HES data, i.e. variable 43 or 44) or prevalent cancer at baseline (based on either self-report or cancer registry) are excluded. | MI/stroke within first 2 years of follow-up are excluded.  Individuals with prevalent heart disease (including angina)/stroke at baseline are excluded (based on self-report or HES data, i.e. variable 43 or 44) | CVD deaths (defined as I200/I259 and I600/I698) within first 2 years of follow-up are excluded.  Individuals with prevalent heart disease (including angina)/stroke at baseline (based on self-report or HES data, i.e. variable 43 or 44) are excluded. | Cancers within first 2 years of follow-up are excluded.  Individuals with prevalent cancer are excluded (based on self-report or cancer registry). | Cancer deaths within first 2 years of follow-up are excluded.  Individuals with prevalent cancer are excluded (based on information from cancer registry or self-report). |
| **Co-variates** | |  |  |  |  |  |
| Model 1 | | Age, sex, ethnicity, urban/rural, area-level deprivation | As for all-cause mortality | As for all-cause mortality | As for all-cause mortality | As for all-cause mortality |
| Model 2 | | Model 1 + education, occupation, income + car access | As for all-cause mortality | As for all-cause mortality | As for all-cause mortality | As for all-cause mortality |
| Model 3 | | Model 2 + fresh fruit, raw vegetables, cooked vegetables, smoking, PA at work, strenuous sport duration, other exercise duration, leisure walking duration, DIY duration, shift work, alcohol, longstanding limiting illness /disability, sleep time (3 categories: <7h, 7-8h, >8h) and screen time (TV and computer viewing combined) | As for all-cause mortality | As for all-cause mortality | As for all-cause mortality | As for all-cause mortality |
| Model 4 | | Model 3 + high BP (self-report), medication for high blood pressure, BMI, medication for high cholesterol, medication for diabetes, diabetes diagnosis (self-report) | As for all-cause mortality | As for all-cause mortality | Model 3 + BMI. | Model 3 + BMI |

* excluding all skin cancers (melanoma and other malignant neoplasms of the skin)
