# Supplementary material for: Using alternatives to the car and risk of all-cause, cardiovascular and cancer mortality
Source: Heart. 2018 May 21;104(21):1749–55. doi: 10.1136/heartjnl-2017-312699 (PMC6241630; doi:10.1136/heartjnl-2017-312699)
Supplement: Supplementary file 2 [file heartjnl-2017-312699supp002.docx]

**Additional file 2: Additional results**

**Table A2:** **Baseline characteristics in those included/excluded from the sample**

|  | **Excluded from the main analysis (N=143854)** | | | **Included in the main analysis (N=358779)** | | |
| --- | --- | --- | --- | --- | --- | --- |
|  | **% missing** | **Mean** | **SD** | **% missing** | **Mean** | **SD** |
| Follow-up time (years) | 0.0 | 7.0 | 1.1 | 0.0 | 7.0 | 1.0 |
| Age (yrs) | 0.0 | 57.3 | 8.1 | 0.0 | 56.2 | 8.1 |
| BMI (kg/m^2^) | 2.2 | 27.6 | 5.0 | 0.0 | 27.4 | 4.7 |
| Strenuous sports duration (mins/wk) | 5.0 | 12.0 | 62.1 | 0.0 | 16.8 | 69.1 |
| Other exercise duration (mins/wk) | 5.0 | 56.0 | 117.9 | 0.0 | 64.2 | 120.1 |
| DIY duration (mins/wk) | 5.0 | 106.4 | 257.2 | 0.0 | 112.3 | 244.8 |
| Leisure walking duration (mins/wk) | 5.0 | 100.3 | 168.2 | 0.0 | 99.3 | 155.3 |
|  |  | **%** | **N** |  | **%** | **N** |
| Sex | 0.0 |  |  | 0.0 |  |  |
| Women |  | 60.0 | 86278 |  | 52.2 | 187184 |
| Men |  | 40.0 | 57576 |  | 47.8 | 171595 |
| Smoking status | 2.1 |  |  | 0.0 |  |  |
| Never |  | 54.0 | 77660 |  | 54.6 | 195937 |
| Previous |  | 32.6 | 46921 |  | 35.2 | 126174 |
| Current |  | 11.3 | 16321 |  | 10.2 | 36668 |
| Ethnicity | 1.9 |  |  | 0.0 |  |  |
| White |  | 89.7 | 129065 |  | 95.8 | 343821 |
| Non-white |  | 8.4 | 12071 |  | 4.2 | 14958 |
| Education | 3.2 |  |  | 0.0 |  |  |
| University degree |  | 24.2 | 34843 |  | 35.2 | 126363 |
| A-Levels |  | 29.8 | 42843 |  | 33.4 | 119840 |
| GCSE or equivalent |  | 16.4 | 23547 |  | 16.7 | 59763 |
| None |  | 26.4 | 37974 |  | 14.7 | 52813 |
| Residential status | 3.5 |  |  | 0.0 |  |  |
| Urban |  | 84.8 | 122021 |  | 85.5 | 306869 |
| Town and fringe |  | 5.8 | 8291 |  | 7.1 | 25583 |
| Rural |  | 5.9 | 8467 |  | 7.3 | 26327 |
| Employment status | 4.0 |  |  | 0.0 |  |  |
| Employed - including voluntary/student work |  | 56.9 | 81834 |  | 61.4 | 220415 |
| Unable to work due to sickness |  | 4.8 | 6855 |  | 3.4 | 12082 |
| Not employed/retired |  | 34.3 | 49405 |  | 35.2 | 126282 |
| Occupation | 12.7 |  |  | 0.0 |  |  |
| Managerial/professional |  | 19.3 | 27782 |  | 41.9 | 150277 |
| Administrative/skilled trades |  | 12.2 | 17608 |  | 15.3 | 54722 |
| Professional/customer services |  | 5.9 | 8533 |  | 6.0 | 21524 |
| Operatives/labourers |  | 6.4 | 9179 |  | 5.8 | 20958 |
| Not applicable (e.g. retired) |  | 43.4 | 62436 |  | 31.0 | 111298 |
| Income | 53.7 |  |  | 0.0 |  |  |
| <£31000 |  | 25.8 | 37045 |  | 46.9 | 168379 |
| £31000-<£52000 |  | 11.2 | 16045 |  | 26.4 | 94747 |
| ≥£52000 |  | 9.4 | 13565 |  | 26.7 | 95653 |
| Number of cars owned | 3.2 |  |  | 0.0 |  |  |
| 0 |  | 11.7 | 16816 |  | 7.6 | 27408 |
| 1 |  | 40.9 | 58821 |  | 41.8 | 149807 |
| 2 or more |  | 44.2 | 63559 |  | 50.6 | 181564 |
| Shift work | 0.5 |  |  | 0.0 |  |  |
| None |  | 90.0 | 129486 |  | 90.0 | 322861 |
| Day only |  | 4.7 | 6762 |  | 4.9 | 17474 |
| Includes nights |  | 4.8 | 6928 |  | 5.1 | 18444 |
| Physical activity in workplace | 14.6 |  |  | 0.0 |  |  |
| Not applicable |  | 39.1 | 56302 |  | 38.6 | 138378 |
| Manual |  | 8.6 | 12368 |  | 7.3 | 26334 |
| Standing/walking/some manual |  | 16.3 | 23397 |  | 18.3 | 65751 |
| Light sedentary |  | 9.1 | 13158 |  | 13.9 | 49891 |
| Sedentary |  | 12.3 | 17636 |  | 21.9 | 78425 |
| Longstanding limiting illness or disability | 8.7 |  |  | 0.0 |  |  |
| No |  | 58.5 | 84144 |  | 68.6 | 246105 |
| Yes |  | 32.9 | 47266 |  | 31.4 | 112674 |

**Table A3:** Prospective associations between travel mode and all-cause, cardiovascular and cancer mortality and incident CVD and cancer for those regularly commuting

|  | | | | **All-cause mortality** | | | | | **Incident CVD** | | **CVD mortality** | | | | | **Incident cancer^†^** | | **Cancer mortality^†^** | | | | |
| --- | --- | --- | --- | --- | --- | --- | --- | --- | --- | --- | --- | --- | --- | --- | --- | --- | --- | --- | --- | --- | --- | --- |
| **Commuting** | | | | **Events** | | **Person years** | | | **Events** | **Person years** | **Events** | **Person years** | | | | **Events** | **Person years** | **Events** | | | **Person years** | |
| Relying exclusively on the car | | | | 1109 | | 767351 | | | 1118 | 809613 | 175 | 814706 | | | | 2704 | 778301 | 737 | | | 792122 | |
| More active patterns of travel | | | | 588 | | 432773 | | | 477 | 456541 | 65 | 458599 | | | | 1442 | 437698 | 366 | | | 444612 | |
|  | | | **N=170511** | | | | | **HR (95%CI)** | **N=180942** | **HR (95%CI)** | **N=181388** | | | **HR (95%CI)** | | **N=174381** | **HR (95%CI)** | | | **N=175877** | **HR (95%CI)** | |
| Model 1 | | | |  | 0.96 (0.86 to 1.06) | | | |  | **0.77 (0.69 to 0.86)** |  | **0.66 (0.49 to 0.89)** | | | |  | 0.99 (0.92 to 1.05) | |  | | 0.90 (0.79 to 1.03) | |
| Model 2 | | | |  | 0.95 (0.85 to 1.06) | | | |  | **0.84 (0.74 to 0.94)** |  | **0.67 (0.49 to 0.91)** | | | |  | 0.99 (0.92 to 1.06) | |  | | 0.93 (0.81 to 1.06) | |
| Model 3 | | | |  | 0.98 (0.88 to 1.10) | | | |  | **0.87 (0.77 to 0.98)** |  | **0.68 (0.50 to 0.93)** | | | |  | 1.00 (0.93 to 1.07) | |  | | 0.96 (0.83 to 1.10) | |
| Model 4 | | | |  | 1.00 (0.89 to 1.12) | | | |  | **0.89 (0.79 to 1.00)** |  | - 1. **(0.51 to 0.95)** | | | |  | 1.00 (0.94 to 1.08) | |  | | 0.97 (0.84 to 1.11) | |
| **Other travel** | | | | **Events** | **Person years** | | | | **Events** | **Person years** | **Events** | **Person years** | | | | **Events** | **Person years** | | **Events** | | | **Person years** |
| Relying exclusively on the car | | | | 818 | 542960 | | | | 781 | 572094 | 124 | 575687 | | | | 1932 | 550434 | | 539 | | | 560120 |
| More active patterns of travel | | | | 881 | 655676 | | | | 813 | 692434 | 114 | 696000 | | | | 2212 | 664003 | | 566 | | | 675010 |
|  | **N=170306** | | | | | | **HR (95%CI)** | | **N=180716** | **HR (95%CI)** | **N=181163** | | | | **HR (95%CI)** | **N=174165** | **HR (95%CI)** | | **N=175656** | | | **HR (95%CI)** |
| Model 1 | |  | | | **0.88 (0.80 to 0.97)** | | | |  | **0.86 (0.78 to 0.95)** |  | | **0.75 (0.58 to 0.97)** | | |  | 0.96 (0.90 to 1.02) | |  | | | **0.86 (0.76 to 0.97)** |
| Model 2 | |  | | | **0.86 (0.78 to 0.95)** | | | |  | **0.90 (0.81 to 1.00)** |  | | **0.75 (0.58 to 0.98)** | | |  | 0.96 (0.90 to 1.02) | |  | | | **0.87 (0.77 to 0.98)** |
| Model 3 | |  | | | 0.91 (0.83 to 1.01) | | | |  | 0.95 (0.85 to 1.05) |  | | 0.77 (0.59 to 1.01) | | |  | 0.98 (0.91 to 1.04) | |  | | | 0.91 (0.81 to 1.03) |
| Model 4 | |  | | | 0.92 (0.83 to 1.02) | | | |  | 0.96 (0.87 to 1.07) |  | | 0.78 (0.60 to 1.02) | | |  | 0.98 (0.92 to 1.05) | |  | | | 0.92 (0.81 to 1.04) |

For non-commuting travel, the reference group is those who use the car for non-commuting travel. For commuting the reference group is those who use the car for commuting. HR: Hazard Ratios; CI: Confidence Interval.

Model 1: age (underlying timescale), sex, ethnicity, urban/rural, area-level deprivation.

Model 2: Model 1 plus education, occupation, household income, cars owned.

Model 3: Model 2 plus fresh fruit, raw vegetables, cooked vegetables, smoking, PA at work, strenuous sport duration, other exercise duration, leisure walking duration, DIY duration, shift work, alcohol consumption, longstanding limiting illness/disability, sleep time, screen time.

Model 4: Model 3 plus hypertension, medication for high blood pressure, BMI, medication for high cholesterol, medication for diabetes, diabetes diagnosis.

**^†^** For cancer outcomes Model 4 adjusted for all variables in Model 3 plus BMI only

**Table A4:** Prospective associations between the commuting and non-commuting travel and all-cause, cardiovascular and cancer mortality and incident CVD and cancer for those regularly commuting

|  | | **HR (95% CI)** | | | | |
| --- | --- | --- | --- | --- | --- | --- |
|  | | **All-cause mortality**  **N=170127** | **Incident CVD**  **N=180533** | **CVD mortality N=180979** | **Incident cancer**  **N=173982** | **Cancer mortality**  **N=175472** |
| Model 1 | |  |  |  |  |  |
| Exclusive use of a car for commuting, more active patterns of travel for non-commuting travel | **0.87 (0.77 to 0.98)** | | 0.91 (0.81 to 1.03) | 0.97 (0.72 to 1.31) | 0.96 (0.89 to 1.04) | 0.87 (0.75 to 1.01) |
| More active patterns of travel for commuting, exclusive use of a car for non-commuting | 0.99 (0.82 to 1.19) | | **0.76 (0.62 to 0.94)** | 1.08 (0.67 to 1.72) | 1.01 (0.90 to 1.14) | 0.95 (0.75 to 1.20) |
| More active patterns of travel for commuting and non-commuting | **0.88 (0.78 to 1.00)** | | **0.74 (0.65 to 0.84)** | **0.55 (0.39 to 0.80)** | 0.96 (0.88 to 1.04) | **0.83 (0.71 to 0.97)** |
| Model 2 |  | |  |  |  |  |
| Exclusive use of a car for commuting, more active patterns of travel for non-commuting travel | **0.86 (0.76 to 0.97)** | | 0.92 (0.81 to 1.04) | 0.96 (0.70 to 1.30) | 0.96 (0.89 to 1.04) | 0.87 (0.75 to 1.01) |
| More active patterns of travel for commuting, exclusive use of a car for non-commuting | 1.01 (0.83 to 1.22) | | **0.81 (0.65 to 1.00)** | 1.10 (0.69 to 1.77) | 1.02 (0.90 to 1.15) | 0.97 (0.77 to 1.24) |
| More active patterns of travel for commuting and non-commuting | **0.86 (0.76 to 0.98)** | | **0.81 (0.70 to 0.93)** | **0.54 (0.37 to 0.80)** | 0.96 (0.88 to 1.04) | **0.84 (0.72 to 0.99)** |
| Model 3 |  | |  |  |  |  |
| Exclusive use of a car for commuting, more active patterns of travel for non-commuting travel | 0.91 (0.80 to 1.03) | | 0.97 (0.86 to 1.09) | 0.99 (0.73 to 1.35) | 0.98 (0.91 to 1.06) | 0.92 (0.79 to 1.06) |
| More active patterns of travel for commuting, exclusive use of a car for non-commuting | 1.04 (0.86 to 1.26) | | 0.85 (0.68 to 1.05) | 1.15 (0.71 to 1.85) | 1.02 (0.91 to 1.16) | 1.01 (0.79 to 1.28) |
| More active patterns of travel for commuting and non-commuting | 0.92 (0.80 to 1.05) | | **0.86 (0.75 to 0.99)** | **0.56 (0.38 to 0.83)** | 0.98 (0.90 to 1.06) | 0.90 (0.76 to 1.06) |
| Model 4 |  | |  |  |  |  |
| Exclusive use of a car for commuting, more active patterns of travel for non-commuting travel | 0.92 (0.81 to 1.04) | | 0.98 (0.87 to 1.11) | 1.00 (0.73 to 1.36) | 0.98 (0.91 to 1.06) | 0.92 (0.79 to 1.07) |
| More active patterns of travel for commuting, exclusive use of a car for non-commuting | 1.05 (0.87 to 1.28) | | 0.86 (0.70 to 1.07) | 1.16 (0.72 to 1.87) | 1.03 (0.91 to 1.16) | 1.01 (0.80 to 1.29) |
| More active patterns of travel for commuting and non-commuting | 0.94 (0.82 to 1.07) | | 0.89 (0.77 to 1.02) | **0.57 (0.39 to 0.85)** | 0.99 (0.91 to 1.07) | 0.91 (0.77 to 1.07) |

HR: Hazard Ratios; CI: Confidence Interval. In models 1-4, the reference group is those who use the car for both commuting and non-commuting travel.

Model 1: age (underlying timescale), sex, ethnicity, urban/rural, area-level deprivation.

Model 2: Model 1 plus education, occupation, household income, cars owned.

Model 3: Model 2 plus fresh fruit, raw vegetables, cooked vegetables, smoking, PA at work, strenuous sport duration, other exercise duration, leisure walking duration, DIY duration, shift work, alcohol consumption, longstanding limiting illness/disability, sleep time, screen time.

Model 4: Model 3 plus hypertension, medication for high blood pressure, BMI, medication for high cholesterol, medication for diabetes, diabetes diagnosis.

**^†^** For cancer outcomes Model 3 adjusted for all variables in Model 3 plus BMI only

**Table A5:** Prospective associations between travel mode and all-cause, cardiovascular and cancer mortality and incident CVD and cancer for those not regularly commuting

|  | **All-cause mortality** | | **Incident CVD** | | | **CVD mortality** | | **Incident cancer^†^** | | **Cancer mortality^†^** | |
| --- | --- | --- | --- | --- | --- | --- | --- | --- | --- | --- | --- |
|  | **Events** | **Person years** | **Events** | **Person years** | | **Events** | **Person years** | **Events** | **Person years** | **Events** | **Person years** |
| Relying exclusively on the car | 1211 | 330672 | 940 | 366984 | | 174 | 370968 | 2215 | 354331 | 798 | 365325 |
| More active patterns of travel | 2245 | 632070 | 1681 | 699439 | | 351 | 706259 | 4001 | 670139 | 1446 | 690701 |
|  | **HR (95%CI)** | | | | | | | | | | |
|  | **N=138352** | | **N=155074** | | **N=138352** | | | **N=149726** | | **N=152222** | |
| Model 1  Model 2  Model 3  Model 4 | **0.88 (0.82 to 0.95)**  **0.84 (0.78 to 0.90)**  **0.92 (0.85 to 0.99)**  **0.92 (0.86 to 0.99)** | | **0.89 (0.82 to 0.96)**  **0.87 (0.80 to 0.95)**  0.93 (0.85 to 1.01)  0.94 (0.87 to 1.03) | | 0.97 (0.80 to 1.16)  0.88 (0.73 to 1.06)  0.96 (0.79 to 1.17)  0.98 (0.81 to 1.20) | | | **0.91 (0.87 to 0.96)**  **0.93 (0.88 to 0.98)**  **0.95 (0.90 to 1.00)**  0.95 (0.90 to 1.01) | | **0.88 (0.81 to 0.97)**  **0.88 (0.81 to 0.97)**  0.94 (0.86 to 1.03)  0.95 (0.86 to 1.04) | |

The reference group is those who use the car for travel. HR: Hazard Ratios; CI: Confidence Interval.

Model 1: age (underlying timescale), sex, ethnicity, urban/rural, area-level deprivation.

Model 2: Model 1 plus education, occupation, household income, cars owned.

Model 3: Model 2 plus fresh fruit, raw vegetables, cooked vegetables, smoking, PA at work, strenuous sport duration, other exercise duration, leisure walking duration, DIY duration, shift work, alcohol consumption, longstanding limiting illness/disability, sleep time, screen time.

Model 4: Model 3 plus hypertension, medication for high blood pressure, BMI, medication for high cholesterol, medication for diabetes, diabetes diagnosis.

**^†^** For cancer outcomes Model 4 adjusted for all variables in Model 3 plus BMI only

**Table A6:** Prospective associations between travel mode and secondary outcomes for those regularly commuting and those not making regular commuting trips

|  | | | **Incident breast cancer** | | | | | | | **Breast cancer mortality** | | | | | **Incident colon cancer** | | | **Colon cancer mortality** | |
| --- | --- | --- | --- | --- | --- | --- | --- | --- | --- | --- | --- | --- | --- | --- | --- | --- | --- | --- | --- |
| **Regular commuters** | | |  | | | | | | |  | | | | |  | | |  | |
| **Commuting** | | | **Events** | | | **Person years** | | | | **Events** | | **Person years** | | | **Events** | | **Person years** | **Events** | **Person years** |
| Relying exclusively on the car | | | 696 | | | 825396 | | | | 37 | | 829719 | | | 283 | | 1007112 | 73 | 1008479 |
| More active patterns of travel | | | 409 | | | 461983 | | | | 23 | | 464452 | | | 152 | | 567569 | 30 | 568229 |
|  | | |  | | | |  | |  | | | |  |  | | |  |  |  |
| Model 1 | | **N=183899** | | | | 1.02 (0.90 to 1.15) | | **N=184398** | | | 1.08 (0.63 to 1.86) | | | | | **N=220443** | 1.04 (0.85 to 1.28) | **N=220587** | 0.84 (0.54 to 1.31) |
| Model 2 | |  | | | | 1.03 (0.90 to 1.17) | |  | | | 0.88 (0.49 to 1.57) | | | | |  | 1.15 (0.91 to 1.45) |  | 0.98 (0.61 to 1.58) |
| Model 3 | |  | | | | 1.03 (0.91 to 1.18) | |  | | | 0.88 (0.49 to 1.58) | | | | |  | 1.17 (0.92 to 1.48) |  | 0.96 (0.59 to 1.57) |
| Model 4 | |  | | | | 1.04 (0.91 to 1.19) | |  | | | 0.89 (0.50 to 1.59) | | | | |  | 1.18 (0.93 to 1.50) |  | 0.95 (0.58 to 1.56) |
| **Non-commuting travel** | | **Events** | | | | **Person years** | | **Events** | | | **Person years** | | | | | **Events** | **Person years** | **Events** | **Person years** |
| Relying exclusively on the car | | 503 | | | | 583187 | | 28 | | | 586189 | | | | | 208 | 709478 | 55 | 710445 |
| More active patterns of travel | | 600 | | | | 702487 | | 32 | | | 706256 | | | | | 228 | 861870 | 50 | 862930 |
|  | | |  | | |  | |  | | |  | | | | |  |  |  |  |
| Model 1 | **N=183661** | | | | | 0.97 (0.86 to 1.10) | | **N=184158** | | | 0.95 (0.57 to 1.59) | | | | | **N=219987** | 0.93 (0.77 to 1.13) | **N=220131** | 0.79 (0.53 to 1.17) |
| Model 2 |  | | | | | 0.98 (0.86 to 1.10) | |  | | | 0.82 (0.48 to 1.40) | | | | |  | 0.95 (0.77 to 1.17) |  | 0.78 (0.51 to 1.18) |
| Model 3 |  | | | | | 1.01 (0.89 to 1.14) | |  | | | 0.84 (0.49 to 1.44) | | | | |  | 0.97 (0.78 to 1.20) |  | 0.79 (0.51 to 1.23) |
| Model 4 |  | | | | | 1.01 (0.90 to 1.15) | |  | | | 0.84 (0.49 to 1.45) | | | | |  | 0.98 (0.79 to 1.22) |  | 0.79 (0.51 to 1.22) |
| **Those not making regular commutes** | | | | | |  | |  | | |  | | | | |  |  |  |  |
| **Non-commuting travel** | | | | | **Events** | **Person years** | | **Events** | | | **Person years** | | | | | **Events** | **Person years** | **Events** | **Person years** |
| Relying exclusively on the car | | | | | 405 | 395898 | | 32 | | | 398494 | | | | | 298 | 621888 | 76 | 623492 |
| More active patterns of travel | | | | | 734 | 744986 | | 43 | | | 749889 | | | | | 626 | 1193579 | 147 | 1196739 |
|  | | | | |  |  | |  | | |  | | | | |  |  |  |  |
| Model 1 | | | | **N=165755** | | 0.92 (0.82 to 1.05) | | **N=166330** | | | 0.69 (0.43 to 1.10) | | | | | **N=259950** | 1.05 (0.91 to 1.21) | **N=260332** | 1.02 (0.77 to 1.35) |
| Model 2 | | | | |  | 0.92 (0.81 to 1.04) | |  | | | 0.66 (0.41 to 1.06) | | | | |  | 1.02 (0.87 to 1.19) |  | 1.00 (0.72 to 1.38) |
| Model 3 | | | | |  | 0.94 (0.82 to 1.06) | |  | | | 0.76 (0.47 to 1.24) | | | | |  | 1.02 (0.86 to 1.20) |  | 1.04 (0.74 to 1.47) |
| Model 4 | | | | |  | 0.95 (0.83 to 1.08) | |  | | | 0.78 (0.48 to 1.27) | | | | |  | 1.02 (0.86 to 1.21) |  | 1.05 (0.75 to 1.48) |
